# Psychological Screening, Standards and Spinal Cord Injury: Introducing Change in NHS England Commissioned Services

**DOI:** 10.3390/jcm12247667

**Published:** 2023-12-13

**Authors:** Jane Duff, Rebecca Ellis, Sally Kaiser, Lucy C Grant

**Affiliations:** 1 Department of Clinical Psychology, National Spinal Injuries Centre, Stoke Mandeville Hospital, Buckinghamshire Healthcare NHS Trust, Aylesbury HP21 8AL, UK; 2 Department of Clinical Health Psychology, Yorkshire Regional Spinal Injuries Centre, Mid Yorkshire NHS Trust, Wakefield WF1 4DG, UK; rebecca.ellis40@nhs.net; 3 Department of Clinical Psychology, Midlands Centre for Spinal Injuries, The Robert Jones and Agnes Hunt Orthopaedic Hospital NHS Foundation Trust, Oswestry SY10 7AG, UK; sally.kaiser@nhs.net

**Keywords:** spinal cord injury, spinal cord disorders, mental health, clinical psychology, rehabilitation, screening, health psychology, quality improvement, psychiatry, ISCoS

## Abstract

Psychologist resourcing across the United Kingdom (UK) spinal cord injury centres (SCICs) varies considerably, which has detrimentally impacted standardising service provision for people with spinal cord injuries/disorders (PwSCI/D) compared with other nations. This paper presents the outcome of a project involving the Spinal Cord Injury Psychology Advisory Group (SCIPAG) and NHS England Clinical Reference Group/SCI transformation groups to agree upon screening and standards and shares data from the National Spinal Injuries Centre (NSIC) and the Yorkshire and Midlands Regional SCICs. Inpatients completed the GAD-7, the PHQ-9, and the short form of the Appraisals of DisAbility: Primary and Secondary Scale (ADAPSSsf), assessing adjustment. A total of 646 participants were included, with 43% scoring above the clinical threshold on at least one of the measures on admission. A subset of 272 participants also completed discharge measures and 42% remained above the threshold on discharge, demonstrating sustained psychological need. This paper provides support for services to move to a screen-and-assessment model supplemented by referral options for those with changing needs or who present with difficulties outside the remit of screening. The findings also support the efficacy of universal screening across the system and consideration of screening and standards for psychological care by the wider psychology community.

## 1. Introduction

There is a wealth of literature that identifies the role and impact that psychological factors can have on the outcome following a spinal cord injury/disorder (SCI/D). This includes, but is not exclusive to, rehabilitation gain and progress [1], surgical responsiveness [2], development of secondary health conditions [3,4], long-term quality of life and morbidity [5]. However, despite this awareness, as well as substantial research and some worldwide (though localised) initiatives, routine and systematic international recommendations for psychological health screening and standards for SCI/D care have been delayed compared with many other physical health conditions [6]. Comparison can be drawn most notably in the services for people who have experienced burns, stroke or cancer. For example, in the UK, The National Burn Care Review Committee recommended screening in 2001, which subsequently led to a pathway identifying “Levels of Psychological Care” for “watchful waiting” (Level 1) and psychological therapy/treatment (Level 2), with later developments including psychoeducation as part of the treatment pathway [7,8,9]. In oncology in the United States, work commenced in 1997, with a direct influence of this being the acknowledgement that many distressed patients are unrecognised and untreated. Pivotally, it was stated that the “management of a patient’s psychological state is vital to the care of every patient at all stages of disease, irrespective of disease site or treatment…In fact, there is no other dimension of cancer that is quite so central to *every* patient” (*sic*) [10] (p. 109). Guidance was published by The National Comprehensive Cancer Network in 2003 [11]. In the UK, the National Institute for Health and Care Excellence (NICE) is the overarching body that approves centralised funding and, consequently, recommends a range of treatment protocols and medication. NICE guidance for improving palliative care cancer services was published in 2004 and included a recommendation to ensure “all patients undergo systematic psychological assessment at key points and have access to appropriate psychological support…[and] a four-level model of screening and professional psychological assessment and intervention” [12] (p. 9).

Similarly, with regard to stroke, the Physical Health and Disability Special Interest Group of the British Psychological Society commenced standard setting and screening in 1999, with publication in 2002 [13]. The guidance identified a service specification, mechanisms for monitoring quality and outcome, and recommended staffing levels. In 2016, the Intercollegiate Stroke Working Party produced the fifth NICE-accredited edition, which provided over 150 pages of comprehensive interdisciplinary guidance and measurement of outcomes, including goal setting, intensity of therapy and self-management. The recommended psychological service was a “matched care model” in which all patients are assessed and provided with needs-based treatment. The “matched care model” is stratified and includes an initial triage so that people commence at the level of treatment needed, which could be the highest and most intense. A further enhancement of this is “the matched collaborative care model”, which includes collaborative goal setting and self-management training. It is different from the “stepped care model” commonly used in mental health services, where people usually commence at the lowest intervention, which tends to be more generic, and is often a group skills-based or online treatment with stepping up in treatment intensity and access to individual therapy if symptoms fail to improve.

The introduction of standards and screening in each of these conditions has led to several developments. In cancer and burns services, there was recognition that culture and the routine of healthcare delivery could enhance or reduce someone’s psychological response, with the development of training and education for multi-disciplinary staff to detect and respond to psychological risk factors. In cancer services, the application of standardised psychometric scales enhanced the understanding of “caseness” and cultural sensitivity, as well as the recognition of psychologist staffing resource limitations [11]. Similarly, in stroke services, standards enabled significant service expansion due to the specificity of the recommendations and the ability of services to review, benchmark and evidence their needs. 

Regarding SCI/D, the 1999 Paralyzed Veterans of America “Spinal Cord Medicine Consumer Guide on Depression” was possibly the first widely available consumer publication aimed at normalising emotional response to injury. Several attempts were made in the intervening years, as outlined in the foreword of the subsequent guidance, to gain funding for the necessary consensus and recommendations for screening, assessment, and treatment regarding anxiety, major depressive disorder, substance use, post-traumatic stress disorder (PTSD)/acute stress disorder and suicide [14]. Although unavoidably delayed, the rigor and detailed nature of the 2021 Clinical Practice Guideline (CPG) provides a comprehensive review of the literature; grades evidence for a recommended treatment; and unites information on psychological, pharmacological and MDT interventions. The CPG dovetails with two other bodies of work from the USA: a series of SCIRehab publications examining the details of psychological treatment and outcome [15] and professional practice standards [16].

Work on the US professional practice standards for psychologists and social workers commenced in 1990. There were various reviews and iterations over time prior to the most recent (2016) version anchoring psychosocial intervention as an inherent part of the biopsychosocial model in rehabilitation. The standards recommended staffing levels and that “psychologists and social workers should be core members of the interdisciplinary treatment team” [16] (p. 135), and the expectation that every inpatient across the lifespan receive psychological health screening and assessment commensurate with the timescale of other team members, including treatment as required. The guidance recommends cognitive behavioural therapy (CBT) and coping effectiveness therapy (CET), rating these as having Level 2 evidence, as well as identifying a range of other interventions. The guidance also recommends a written plan for psychosocial review after discharge and the need for follow-up outpatient screening across the lifespan.

In Australia, psychosocial care guidelines for New South Wales State SCI services were first published in 2008, with later editions in 2013 and 2023 [17,18]. As with the US guidance, a comprehensive psychological assessment for all inpatients was recommended, with a timescale of within 5 days of admission to rehabilitation. The guidance mandated that treatment be provided as required, with CBT being highlighted as an evidence-based intervention and that a psychological review occur prior to discharge, with mood screenings 6 and 12 months after returning to community living. The guidance was accompanied in 2016 by an “Emotional Wellbeing Toolkit: A clinicians guide to working with SCI” which provided measures of psychological screening for mood, PTSD, pain, psychosis, alcohol and substance use, with accompanying clinician tips for these and other concerns, such as suicidality and self-harm, challenging behaviour, traumatic brain injury and dementia [19]. A key element of the toolkit was the recognition that psychosocial care is “everyone’s business” [19] (p. 1) and inherent within every healthcare clinician’s role in SCI/D.

Psychosocial rehabilitation guidelines, which integrate peer counselling as an intervention alongside healthcare clinicians, were published by the Asian Spinal Cord Network in 2015 [20]. Crucially, the guidelines also identified the likelihood of variable provision because of resource limitations and recommended the provision of psychoeducational material about adjustment as an intervention in the absence of peer counsellors or healthcare clinicians with a psychosocial role. The guidance is partly based on a “stepped care model”, with the peer counsellor identifying the need for formal individual psychological assessment and treatment. The family system of the PwSCI/D and their needs are recognised as part of the intervention in Levels 3 and 4, and the guidance names the importance of integrating sexual counselling as routine.

The Netherlands commenced psychological screening in 2018, with inpatients being administered a range of brief measures within two weeks of admission as an inpatient or on commencing an outpatient rehabilitation programme. The programme aimed to evaluate 80% of the people identified; however, an audit found that due to staffing constraints and logistics, only 64% of patients completed the measures [21]. Developed as part of the screening, the programme adopted a national approach of flagging those needing follow-up, with patient responses on each measure being RAG (red, amber, green) rated. Several benefits of screening were identified: timely recognition of psychological problems; screening measures, as administered by a psychologist, providing an introduction to broader psychosocial issues regarding self-efficacy and resilience rather than just a pathological focus; screening enabling more targeted treatment and use of personnel; and, of perhaps greatest significance, increased identification and recognition of psychological issues amongst other rehabilitation team members [21].

Despite the evidenced progress of these individual nations, international agreement on psychological screening and the psychological measures to use has been severely delayed compared with other areas of care for PwSCI/D. The delay has no doubt been impacted by the substantial rigor and time required for such an initiative and the absence of resourcing, as well as the lack of a Psychosocial Special Interest Group (SIG) within the International Spinal Cord Society (ISCoS) until 2018, to co-ordinate and champion the need. However, progress has been swift since the inception of the SIG, with the Basic Psychology Data Set screening recommendations being published in 2023 [22], and work having already commenced on the Advanced Data Set. 

The absence of a systematised international consensus on screening and standards of psychological care has significantly impeded psychosocial care in some nations. One of these has been the UK, with a report on SCICs in England in 2023 showing huge differences in services and psychologist-to-inpatient ratios varying from 1:15 to 1:100 [23], with the lowest provision in Sheffield, Salisbury, Southport and the National Spinal Injuries Centre (NSIC) at Stoke Mandeville. The 2008 rehabilitation standards include one reference regarding the need to assess depressed mood [24] but, in contrast to many of the initiatives of other nations, did not include an expectation that assessment would be universal, that a psychologist should be included as a core member of the rehabilitation team, or that there be a timescale for screening and assessment. The current SCI/D service specification lists psychometric assessment at mobilisation and again at discharge as one of the key indicators and includes a reference to the employment of clinical psychologists with specialist expertise in SCI/D care and their involvement in MDT and treatment decision-making meetings [25,26]. However, this is the limit of the scope, and it does not specify the nature of the psychometric screen or assessment. 

In 2014, the first author was part of a working group which agreed national psychology screening measures. These were not implemented due to the delayed revision of the NHS England Database; however, this enabled the UK and Ireland Spinal Cord Injury Psychology Advisory Group (SCIPAG) to revise, in 2019, the mood screen from the Hospital Anxiety and Depression Scale [27] to the Patient Health Questionnaire-9 for depression [28] and Generalised Anxiety Disorder-7 [29], with each NHS England commissioned SCIC agreeing to opt in where able and screen on admission and discharge from April 2021. Alongside this development, in March 2020, a small working group of SCIPAG, which comprised the first author and third author, clinical psychologists working in the Yorkshire and Welsh SCICs, and two people with lived experience from the Back Up Trust and Spinal Injuries Association, commenced work on psychological care standards.

There is a significant capacity gap for access to specialised SCI/D rehabilitation in the SCICs commissioned by NHS England. The 2021 annual Database report showed that only 36% of PwSCI/D were admitted to inpatient rehabilitation [30]. The trend of roughly one-third of newly injured PwSCI/D receiving rehabilitation through outpatient services of the SCIC and one-third through another NHS provider (most commonly a district general hospital or neurorehabilitation service) has been present for some time. However, these figures are likely to be an underestimate of need, given the Database captures referrals rather than nationally mandated reporting of PwSCI/D incidence. The UK Spinal Injuries Association estimates there to be seven new PwSCI/Ds each day and 2500 injuries per annum [31]. In 2020, in part to address the access gap, NHS England and SCI/D CRG broadened the scope of SCICs to include responsibility for all PwSCI/D within each service’s catchment area and developed seven transformation workstreams to set standards and recommendations across the care pathway. The first author, as the chair of SCIPAG and a member of the SCI/D Clinical Reference Group (CRG, which oversees service provision for NHS England), led the workstream for psychological and mental health needs [23], which ratified the previously agreed screening measures and introduced several other recommendations; the first author also contributed to this work the psychology standards developed as part of SCIPAG (Appendix A) [32].

At the inception of the workstreams in October 2020, most of the psychology services within the eight NHS England commissioned SCICs did not have the staffing resources to assess every patient on admission and/or discharge, and the dominant model was based on referral rather than screening. To assess the referral versus screening gap and evidence the need for a changed model within its own service, the NSIC at Stoke Mandeville completed a retrospective audit of 166 inpatients between November 2020 and April 2022 and compared referral with screening data on the PHQ-9 [28]; the GAD-7 [29]; and the short form of the Appraisals of DisAbility Primary and Secondary Scale (ADAPSSsf), which measures adjustment [33]. Thirty percent of inpatients had a positive screen on at least one of these measures at admission; by discharge, 27% of the same inpatients had a positive screen and, crucially, had not been referred to the psychology service during their admission [34]. Each of the centres involved in this research had a slightly different service model. The NSIC at Stoke Mandeville had a 33:1 patient-to-clinical-psychologist ratio with a referral model and “watchful wait” monitoring through the MDT for those who screened in but had not been referred. The Yorkshire Regional Spinal Injuries Centre (YRSIC) had a 27:1 ratio and aimed to screen and assess every patient within 2 weeks of admission; however, after the implementation of screening, this centre experienced staffing difficulties and was operating on a referral model for the period of this study. The Midlands Centre for Spinal Injuries (MCSI) had a 29:1 ratio and either used MDT screening or the screen was combined with a psychological assessment with screening over the first 4 weeks of an inpatient’s admission but did assess all first-time admissions for rehabilitation.

As referenced earlier, screening has been implemented by many psychology services as a means of discerning initial needs, augmented with a referral process in recognition that adjustment is not a linear process [4,14,35]. One of the influencers for the rise of screening across services rather than a clinician-led referral has been the recognition that “physicians continue to use personal experience as part of their decision-making process and are subject to a wide range of influences, despite the recent emphasis on the use of EBM [evidence-based medicine]” [36] (p. 184) and that “non-clinical influences on decision-making may be the most important, and up to now largely unrecognized obstacle to the practice of EBM”, with a range of patient-related factors, as well as unconscious bias being present [36] (p. 179). Although the above references physician decision making, it is well known that all healthcare clinicians need to be sensitive to and aware of the risk of a uni-professional approach. A survey of MDT staff training needs was conducted by the NSIC at Stoke Mandeville: seven NHS hospitals that refer into the service, as well as private providers and third-sector organisations. Across 13/15 key areas of psychological care, respondents indicated low confidence and the need for training to develop their awareness of patient’s psychological needs [37]. It was knowledge of these issues that influenced SCIPAG to implement the option to screen to augment the dominant MDT referral model within services in April 2021.

The psychology standards and psychological and mental health workstream were significantly influenced by the body of international work outlined earlier and aimed to incorporate the key learnings and initiatives within an evidence-based, system-wide model for NHS England SCI/D services. Appendix A, Supplementary Tables S1 and S2, and Supplementary Figure S1 reference the details of the recommendations for system-wide change: these include agreement regarding standards; adoption of a screening and matched collaborative care pathway (in which all PwSCI/D receive assessment and different levels of intervention based on their level of psychological complexity); an MDT curriculum (for enhancing knowledge and recognition of psychological care needs); and identified several developments, most crucially the need for workforce parity across SCICs to achieve implementation.

In light of the recent advances in standardising psychology provision for PwSCI/D, the current study sought to compare the introduction of screening across three NHS England commissioned SCICs: NSIC at Stoke Mandeville, MCSI and YRSIC. This study aimed to understand the incidence of psychological needs on admission and discharge from inpatient rehabilitation and to consider, in light of the standards, any recommendations for services that are working within a referral model. It was also hoped that the results could be used to inform the staffing resources needed to embed the standards and service changes within the participating centres and across all UK SCICs and to raise awareness of the psychological screening needs for the two-thirds of PwSCI/D not admitted to specialist SCIC rehabilitation. To aid the reader’s understanding of the NHS in England, acronyms and a brief contextual explanation can be found in Abbreviation.

## 2. Materials and Methods

### 2.1. Study Design and Procedure

This study employed a multicentre retrospective cohort design. Two centres, namely, the NSIC and YRSIC, collected data via a “screen and refer” model, in which all inpatients received a psychometric screen at admission, but only those referred to the centres’ respective psychology teams received a psychological intervention. The remaining centre, namely, the MCSI, operated an “assessment and screen” model, in which all inpatients were provided with a psychological assessment, which included psychometric screening as part of this.

Data collected at the NSIC were extracted from the psychological health domain of the Stoke Mandeville Spinal Needs Assessment Checklist (SMS-NAC), which is a self-report, MDT-administered, validated measure [38] used to assess inpatients’ knowledge, skills, and physical or verbal independence across 10 domains of rehabilitation for SCI/D. The SMS-NAC should be administered within 2 weeks of admission to the rehabilitation centre to aid goal setting, and within four weeks of discharge to provide an outcome measure. Data from the NSIC were collected between January 2020 and September 2023 for all inpatients with SCI/D who had completed an adult version of the SMS-NAC at admission. Data from the YRSIC were collected between January 2019 and September 2023 as part of the Psychology Service screening proforma administered to all inpatients with SCI/D; the screening ranged from 2–67 days, with a mean time of 19 days for this study sample.

Data from the MCSI were collected between October 2021 and September 2023. Prior to mid-February 2023, demographic and psychometric information were extracted from the relevant sections of the adult version of the SMS-NAC, which was administered by a member of the inpatient’s MDT. After this date, psychometric measures were instead administered by the centre’s assistant psychologist to aid the completion rate. Measures were administered within three weeks of inpatients’ admission to the SCIC.

### 2.2. Participants

PwSCI/D with an age at injury of at least 16 years who were admitted to an SCIC for first-time rehabilitation were eligible for inclusion in this study. Individuals with significant cognitive or language impairment who were unable to understand the screening measures were excluded. Overall, 759 participants across the 3 centres met inclusion criteria, while 113 did not complete screening measures and were excluded from this study. Of the total number of included participants with admission measures (*n* = 646), 272 had also completed discharge measures. Where necessary, incomplete demographic information was supplemented by checking participants’ electronic patient notes.

### 2.3. Ethics

Retrospective data from the 3 SCICs were collected individually; however, participants’ identifiable information was removed from their datasets by authors S.K. and R.E. prior to sending it to authors L.C.G. and J.D. for data collation and analyses. De-identified demographic information and psychometric data (comprising total scores on the PHQ-9 [28], the GAD-7 [29] and the short form ADAPSSsf [33]) were shared via secure email.

Participant consent was not required due to the data being collected as part of standard clinical care. All relevant approvals were completed prior to data transfer to ensure anonymous and ethical data sharing. The NHS England Health Research Authority provided approval for anonymised data sharing, and all research and governmental protocols were adhered to for the duration of this study.

### 2.4. Study Variables

Psychometric data for analyses comprised the PHQ-9, GAD-7 and ADAPSSsf. The PHQ-9 was used to screen for the presence of depressive symptoms at admission and prior to discharge. Participants indicate on a 4-point Likert scale, from 0 (“Not at all”) to 3 (“Nearly everyday”), how often over the previous two weeks they had experienced each item. The total score, ranging from 0 to 27, is calculated by summing all items. Scores of 0–4 indicate subclinical depression, scores of 5–9 indicate mild depression, scores of 10–14 suggest moderate depression, scores 15–19 suggest moderately severe depression and scores of 20–27 indicate severe depression. The PHQ-9 has been validated for use in SCI/D inpatient rehabilitation and demonstrates good diagnostic accuracy and reliability [39,40].

The GAD-7 measure was used to assess the presence of anxiety symptoms. As with the PHQ-9, participants indicate how often over the last two weeks they had experienced each item using a 4-point Likert scale. The total score, ranging from 0 to 21, is calculated by summing all items. Total scores can be subdivided into categories depending on severity, with 0–4 indicating sub-clinical anxiety, scores of 5–9 suggesting mild anxiety, scores of 10–14 suggesting moderate anxiety and scores of 15–21 indicating severe anxiety. The GAD-7 has been validated for use in primary health populations [41], including good validity and reliability for use in the SCI/D population [42,43].

The ADAPSSsf was used to assess participants’ appraisals of their injury and provide an indication of adjustment to SCI/D. The ADAPSSsf consists of 6 items, 3 of which relate to negative appraisals and are included in the “Loss/Catastrophic Negativity” subscale, and 3 which relate to positive appraisals and are included in the “Resilience” subscale. “Loss” items are measured on a 6-point Likert scale from 1 (“Strongly Disagree”) to 6 (“Strongly Agree”), where higher scores indicate a greater sense of loss and more negative appraisal of injury. “Resilience” items are reverse scored, such that higher scores indicate less resilience and more negative appraisal of injury. A total score, ranging from 6 to 36, is calculated by summing each item. The ADAPSSsf shows good validity and reliability for use in SCI/D [33,40].

In line with recommendations from the literature, scores were considered above the threshold if they were ≥11 on the PHQ-9 [39], ≥8 on the GAD-7 [43] and ≥22 on the ADAPSSsf [33]. For each measure, scoring above the threshold indicates that a clinician-led mental health assessment is required to determine a diagnosis. Participants with missing responses on some of the psychometric measures were still included in the analyses as long as at least one of the measures had been fully completed.

### 2.5. Coding and Statistical Analyses

Minor differences existed between the samples in relation to the demographic information that was collected and recorded. De-identified data from the MCSI and YRSIC were therefore coded in line with the NSIC at Stoke Mandeville sample to ensure consistency and homogeneity. Demographic data not reported by all three samples were removed from the analysis. 

For categorical data, chi-square tests of association were performed to compare the demographics between the 3 samples, as well as between participants scoring above compared with below the threshold on the PHQ-9, the GAD-7 and the ADAPSSsf. For continuous data, one-way ANOVAs were used to evaluate whether differences existed for age at injury and time since injury between the samples, as well as for participants who scored above compared with below the threshold on the PHQ-9, the GAD-7 and the ADAPSSsf. Calculations were conducted using all participants’ admission data in addition to the participants with both admission and discharge data. 

Additional analyses were conducted using a subgroup of participants that completed the admission and discharge measures to compare within-sample psychometrics at each time point. Paired-sample *t*-tests were used to determine whether mean differences existed between participants’ admission and discharge scores for all screening measures. Finally, multiple linear regression analysis was used to examine whether the score at admission or demographic variables were predictive of the score at discharge for each of the measures. 

Prior to commencing the analyses, statistical assumptions were first tested to ensure the reliability and validity of the results. For all statistical tests, IBM SPSS Statistics for Windows Version 17.0 was used, and the significance level was set at *p* < 0.01. 

## 3. Results

### 3.1. Admission Data

**Figure 1:**
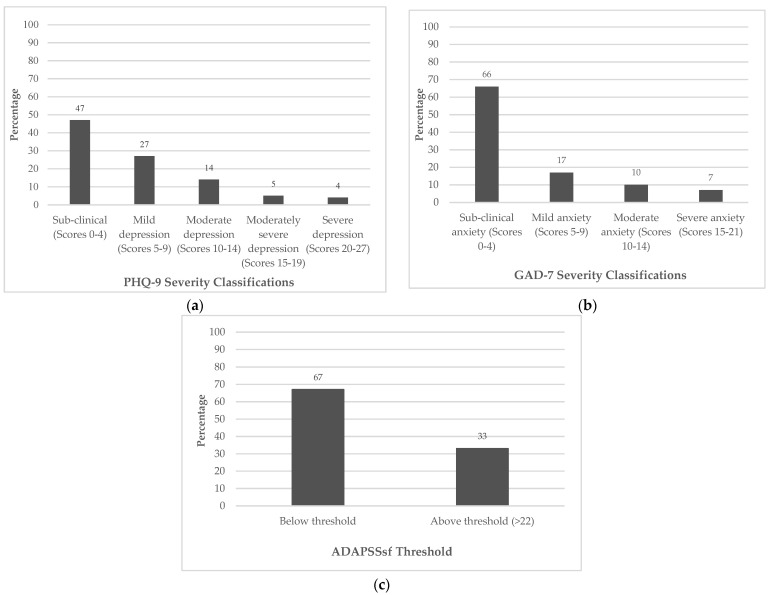
Combined sample percentage severity classifications at admission only for (**a**) PHQ-9, (**b**) GAD-7 and (**c**) ADAPSSsf.

Demographics and descriptive statistics for all participants are presented in Table 1. All participants completed at least one of the screening measures on admission. Forty-three percent (*n* = 281) of participants demonstrated psychological need by scoring above the threshold on at least one of the measures. Admission severity classifications for the PHQ-9, GAD-7 and ADAPSSsf are presented in Figure 1a–c. The psychometric properties for all three measures are shown in Table 2.

When comparing demographics across samples, there was a small overrepresentation of individuals identifying as “White” ethnicity in the YRSIC sample (χ^2^(10) = 50.28, *p* < 0.01, *V* = 0.197). Additionally, there was a small underrepresentation of traumatic injuries (χ^2^(2) = 16.63, *p* < 0.01, *V* = 0.160) and a small-to-moderate underrepresentation of incomplete ASIA D injuries (χ^2^(6) = 60.26, *p* < 0.01, *V* = 0.216) in the YRSIC sample. The YRSIC sample also had a significantly shorter time since injury (*F*(1,2) = 13.67, *p* < 0.01), while the NSIC sample was significantly younger at injury compared with the other samples (*F*(1,2) = 5.66, *p* = 0.004).

A small overrepresentation of females scoring above the threshold on both the PHQ-9 (χ^2^(1) = 8.02, *p* = 0.005, *V* = 0.113) and GAD-7 (χ^2^(1) = 7.31, *p* = 0.007, *V* = 0.107) was observed. Individuals scoring above the threshold on the PHQ-9 (*F*(1630) = 6.73, *p* = 0.01) were significantly younger at injury (*M_above_* = 52.85, *M_below_* = 57.22); however, no significant differences were found in either age at injury or time since injury for the GAD-7 or ADAPSSsf. No other significant differences were observed when comparing demographics for those above compared with below the threshold across the measures with respect to sex, ethnicity, cause of injury or level of injury. 

### 3.2. Subgroup with Admission and Discharge Data

From the total sample, a subgroup of 272 participants had both admission and discharge data. When comparing the demographics of this subgroup to participants with only admission data, the only significant difference observed was for the level of injury, in which there was an overrepresentation in the subgroup individuals with paraplegic (A/B/C) injuries (χ^2^(3) = 15.44, *p* = 0.001, V = 0.155). 

**Figure 2:**
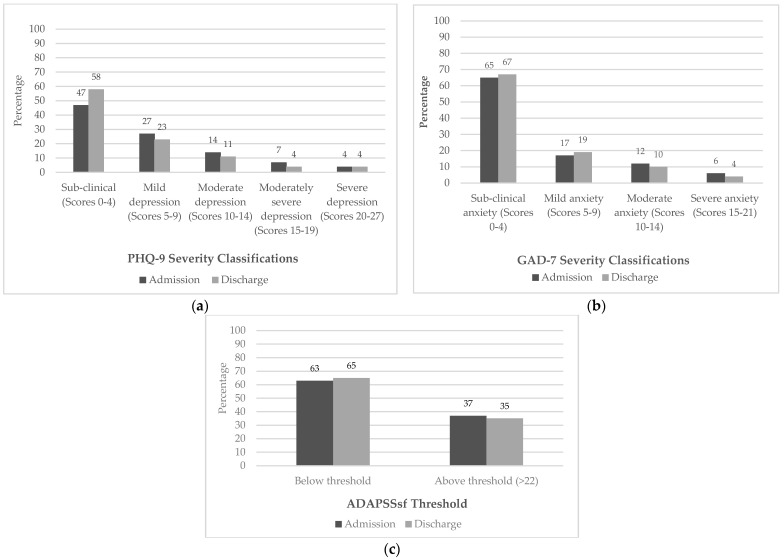
Combined sample percentage severity classifications at admission and discharge for (**a**) PHQ-9, (**b**) GAD-7 and (**c**) ADAPSSsf.

At admission, 46% (*n* = 124) of participants with admission and discharge data demonstrated psychological need by scoring above the threshold on at least one of the screening measures. By discharge, 42% (*n* = 115) still scored above the threshold on at least one of the measures. The total scores for those with both admission and discharge measures across samples are presented in Table 3. The admission and discharge severity classifications for the PHQ-9 and GAD-7 are presented in Table 4 and Figure 2a–c. Psychometric properties for all three measures are shown in Table 5.

For the combined sample, there was a small underrepresentation of people with incomplete ASIA D injuries scoring above the GAD-7 threshold at discharge (χ^2^(2) = 11.90, *p* = 0.003, *V* = 0.210). However, there were no other significant differences for those scoring above compared with below the threshold for either the PHQ-9, GAD-7 or ADAPSSsf with respect to sex, ethnicity or cause of injury.

There were no significant differences in age at injury nor time since injury between the combined samples. However, participants scoring above the threshold at admission on the GAD-7 had a significantly longer time since injury (*M_above_* = 0.48, *M_below_* = 0.33), (*F*(1265) = 8.54, *p* = 0.004). Those scoring above threshold on the PHQ-9 at discharge were significantly younger at injury (*M_above_* = 48.71, *M_below_* = 57.45), (*F*(1268) = 9.01, *p* = 0.003). Similarly, those scoring above threshold on the GAD-7 at discharge had a significantly younger age at injury (*M_above_* = 49.48, *M_below_* = 57.33), (*F*(1267) = 8.23, *p* = 0.004).

The mean PHQ-9 score at admission (*M* = 6.33) was significantly greater than the mean score at discharge (*M* = 5.19), (*t* = 3.36, *p* = 0.001). However, there were no significant mean differences at admission compared with discharge for either the GAD-7 or the ADAPSSsf.

### 3.3. Regression Analyses

For the subgroup that contained admission and discharge measures, a multiple linear regression was calculated to determine whether the admission psychometric score or any demographic variables were predictive of the discharge score (Table 6). A priori power analysis indicated that a multiple regression with seven predictor variables would require a sample size of 142 to achieve a medium effect size of 0.15 with a significant criterion of ɑ < 0.01 and power = 0.80. Thus, the obtained sample size of subgroup participants (*n* = 272) exceeded this minimum requirement.

A higher admission score (*M* = 6.32, *SD* = 5.81) for the PHQ-9, higher level of injury (*Mode* = paraplegic A/B/C) and younger age at injury (*M* = 56.28, *SD* = 16.90) were significantly associated with a higher discharge score and explained approximately 38% of the variance (*F*(7257) = 18.186, *p* < 0.001, *R*^2^ = 0.331). Similarly, the multiple regression for the GAD-7 was significant, with a higher admission score (*M* = 3.80, *SD* = 4.88), younger age at injury (*M =* 56.14, *SD* = 16.80) and higher level of injury (*Mode* = paraplegic A/B/C) predicting a higher discharge score and explained 38% of the variance (*F*(7256) = 22.813, *p* < 0.001, *R*^2^ = 0.384). The strongest relationship was found for the ADAPSSsf, in which only the admission score (*M* = 19.49, *SD* = 6.76) explained 39% of the variance in the discharge score (*F*(7256) = 23.760, *p* < 0.001, *R*^2^ = 0.394).

## 4. Discussion

This study aimed to understand the incidence of psychological need following PwSCI/D’s admission to specialist SCIC rehabilitation and at discharge. Additionally, this study sought to consider whether the current referral model used across most of the SCICs commissioned by NHS England should remain or whether all services should move to a mixed model of screening and referral, with screening being incorporated within routine psychological assessment on admission and intervention aligned with the matched collaborative care pathway. 

A key finding was that between 43 and 46% (depending on whether the combined or subset data were used) of inpatients were above the threshold on at least one of the screening measures, indicating their psychological needs following admission to an SCIC. This was slightly higher than a prior study, which found that 32% of participants scored above the threshold on at least one measure [1]; however, the current study used the PHQ-9 and GAD-7 rather than the HADS and included data from three rather than one SCIC. Focussing specifically on the PHQ-9 and the GAD-7, 23% of individuals scored above the threshold on each measure, which very strongly supported the 22% occurrence of above-threshold symptoms of depression reported across a meta-analysis of 21 studies [44], and 21% occurrence for above threshold symptoms of anxiety in a cross-cultural combined inpatient and community study [43]. Additionally, and comparative to the current retrospective findings, a prospective study tracking inpatients from admission to discharge and through to community living found that the rate of any diagnosed mental disorder was 21.8% at admission and 17.3% at discharge from rehabilitation [45]. 

Compared with the measures that screen for mood, a higher occurrence of psychological need was evidenced for the ADAPSSsf, with 35% of individuals presenting with suspected adjustment difficulties. The inclusion of the ADAPSSsf in this study enhanced and underlined the fact that “the absence of mood disturbance is not necessarily an indicator of adjustment to SCI/D, as this is a complex process that develops as the person experiences new events and understanding of their condition and can take substantial time following transition into the community” [46] (“Interventions for Mental Health Disorders” section). This suggests that to best support the psychological health of PwSCI/D, screening must go beyond merely considering mood by recognising the significance of adjustment following injury and the complex and ever-developing interplay of each. Indeed, research by Guest et al. (2015) argued that even individuals who demonstrate resilience nevertheless continue to face significant challenges of daily living and adjustment to severe injury and its associated lifelong impairment [47]. Echoing the 35% estimated occurrence of adjustment problems in the current sample, Guest and colleagues (2015) similarly suggested that “a large minority of participants (just over 30%) remain highly vulnerable to problems such as maladaptive coping, hopelessness, and negativity in the longer term” [47] (p. 685).

A crucial outcome from this study was that admission scores on all three measures were predictive of the scores at discharge, suggesting that individuals’ degree of psychological need upon entry to an SCIC will influence psychological health outcomes during their rehabilitation and through to discharge. This strongly supports the recommendations for SCICs and other providers to include early and routine psychometric screening as part of the psychological assessment and alongside referral to ensure individuals with psychological needs are recognised. The findings are augmented by previous research suggesting poorer physical rehabilitation outcomes if psychological needs are unrecognised [1], as well as early mortality and long-term adjustment difficulties from symptoms of depression and anxiety at 12 weeks following injury [5,48].

There was a significant reduction in PHQ-9 scores by discharge, which is similar to the findings by Craig et al. (2015) [45]. However, Kennedy et al.’s (2016) longitudinal study found that symptoms of depression and anxiety increased as discharge approached [5], and indeed the current study also revealed that individuals scoring above the PHQ-9 threshold at admission had higher mean scores at discharge. It is important to note the different contextual framework from Kennedy et al. (2016), whose discharge data from an SCIC were collected prior to 1995. Since this date, SCICs have seen the introduction of surgical intervention rather than conservative management, as well as recent demographic changes in the representation of “non-traumatic injuries”, which may reflect some differences in results. A limiting factor in the current study was that psychological intervention was unable to be controlled for during the analyses, which may have accounted for the decrease in depression symptoms by discharge. However, if this were solely responsible for the change in scores, a significant reduction in the scores for the GAD-7 and ADAPSSsf might also have been expected. It is important to acknowledge that discharge from rehabilitation occurs at a time when a physical outcome is achieved rather than guided by psychological variables and that psychological adjustment often takes much longer and can be significantly delayed and influenced by someone’s understanding of their condition and needs following the resumption of community living [46]. This underlines the importance of screening at regular follow-up reviews, as recommended in the USA [14], Australian [19] and current study’s standards (Appendix A) and again reiterates the complexity of psychological need following a spinal cord injury, i.e., it encompasses more than simply the presence versus absence of mood disorder and very rarely follows a linear process. Indeed, the research on trajectories following spinal cord injury provides insight into the varying presentations, with Craig et al. (2019), Bombardier et al. (2021), Bonnano et al. (2012) and van Leeuwen et al. (2012) identifying different pathways for symptoms of depression and anxiety, including a “delayed” pathway in which individuals initially present below the threshold for depression or anxiety but deteriorate to clinical levels over time [4,15,36,49]. A previous study conducted at the NSIC at Stoke Mandeville found that although the patients’ moods and ADAPSSsf scores improved by discharge, there was a deterioration in both at the 18-month follow-up [50]. 

Also identified was that those scoring above the threshold on the PHQ-9 and the GAD-7 at discharge were significantly younger at injury and that younger age at injury was predictive of psychometric measures at discharge. While this only accounted for an approximately 10-year mean age difference (48 years compared with 57 years), this finding may be useful to identify individuals at greater risk of mood disturbance. Indeed, prior research identified an inverse “U-shaped curve” regarding age and depression symptoms in individuals both with and without disability such that middle-aged individuals report more severe depression symptoms than either younger or older individuals [51]. As such, sustaining an injury when approaching middle age compared with later may facilitate or exacerbate depression symptoms in an already at-risk population. Another theory is that individuals with a younger age at injury could be behind their older counterparts in relation to employment, finances, and retirement planning, and therefore may be less prepared for such an adjustment and more susceptible to low mood disturbance. Hirsh et al. (2009) found that PwSCI/D aged between 45 and 54 were more likely to be employed following injury compared with the group aged between 55 and 64, which perhaps lends support to the theory that early middle-aged individuals still rely on the financial security of employment prior to retirement [52]. 

A finding specific to anxiety in the current study was that the participants who scored above the threshold at admission on the GAD-7 had a significantly longer time since injury. The major trauma pathway model states that inpatients should be admitted to SCIC rehabilitation about 1–2 weeks after injury, whereas the current sample had a delay of between 2 and 4 months, which can be understood in the context of the NHS England annual report regarding capacity issues discussed in the introduction. From this study, it is unclear when symptoms of anxiety developed and whether the symptoms were associated with a delay in admission. Systematic screening prior to SCIC admission by major trauma centres, which is one of the recommendations of the standards (Appendix A), would help to illuminate this. Anecdotal accounts from inpatients after SCIC admission and major trauma centre colleagues indicate concerns from inpatients about whether specialist SCIC services would be able to be accessed at all and a sense from the MDT in major trauma centres that inpatients are unable to fully engage in therapies as they wait for the highly prized specialised rehabilitation, as well as an assumption that patients express improved functional outcome from admission to such services, all of which could account for the results found in this study.

As identified in the introduction, screening in other conditions has led to pathway and service developments, including the identification of the need for an increased workforce [53]. In the short-term, screening in resource-constrained services may help to support more effective deployment of psychological resources. However, as commented, this would need to include screening for other variables, such as appraisals, as identified in the current study, as well as a range of other factors, rather than an over-reliance on mood measures to identify adjustment difficulties. Alongside this should be recognition that a range of psychological interventions are needed to face the challenge of such a life-changing event as an SCI/D [54]. Nevertheless, screening and retrospective analysis of referral data [34] have enabled the NSIC at Stoke Mandeville to engage in a “watchful wait” for those who were above the clinical threshold on either the PHQ-9, GAD-7 or ADAPSSsf, allowing for the mitigation of service constraints by providing group-based psychoeducation and through discussion of management and support strategies with MDT colleagues. More recently, the service has taken this a step further and aims to commence a “triage” for all admissions to augment screening and help identify those most in need of psychological assessment [55]. However, there is an accompanying acknowledgement that because of resource constraints, the likely corollary would be the reduction in the frequency of treatment sessions for those identified. 

This study also highlights the variation in service provision across the three SCICs involved despite the introduction of screening, which emphasises the need for standards relating to psychological assessment and treatment, and the embedding of these within clinical practice to accompany the introduction of screening. The introduction to this paper identified the need for international rehabilitation standards and the consequent impact on psychological care. The World Health Organisation 2030 Package of Rehabilitation Interventions, which outlines a range of rehabilitation standards, including psychological guidance, for services and health ministries across the world, will significantly contribute to future service development and parity [56]. The systematic introduction of screening will aid many resource-constrained services to evidence the psychological needs and impact on service provision for PwSCI/D. This may include, as two of the SCICs in the current study demonstrated, MDT colleagues administering psychological health screening. As identified in the Netherlands, screening enhanced non-psychologist rehabilitation members’ appreciation of psychological issues [21]. Bombardier et al.’s (2021) comprehensive overview of screening and intervention includes guidance regarding how MDT colleagues can structure rehabilitation for those identified with mood difficulties and are highly applicable when qualified psychological resources are lacking [14].

### Limitations and Suggestions for Future Research

The current study sought to increase the generalisability of findings by examining data from three SCICs in England. Although there were some demographic differences between the samples, overall, these were small and limited in influence due to the large sample size. However, there was a significant amount of missing data for ethnicity in the NSIC and MCSI samples, which may have contributed to the findings of over-representation of “White” participants in the YRSIC sample. The high proportion of missing data may have also contributed to the lack of significant variation accounted for in discharge psychometric scores. This absence is concerning in several regards in relation to the efficacy of screening. In a study assessing rehabilitation outcome using the SMS-NAC, skills in the domains of physical healthcare and psychological healthcare were self-rated as lower for “Black” than “White” individuals on admission, indicating lesser self-perceived levels of knowledge and independence in these areas [57]. This finding was maintained at discharge, with the addition of lowered self-rated outcomes in community preparation for “Black” individuals. Outcomes on discharge for “White” individuals were also self-rated as significantly higher in the physical healthcare domain than those who identified as “Asian”. Potential differences between ethnic groups are, therefore, of significant importance for both clinicians and researchers to consider in relation to rehabilitation provision and further emphasises the need for psychological screening and routine assessment to identify where issues may reduce someone’s ability to engage and participate and impact their outcome.

The combined datasets across SCICs resulted in a large sample size. However, the subgroup with admission and discharge data was restricted in number due to a large proportion of missing discharge measures which led to difference within the aetiology of injury in the MCSI and YRSIC samples compared to national data. However, the proportions of traumatic and non-traumatic injuries in the combined sample were consistent with the annual report data, and the comparable occurrence of psychological need between the combined and sub-dataset of admission and discharge, as well as with prior research, should restore confidence in the results. Nonetheless, the reduction in completed measures at discharge emphasises the limited workforce capacity across SCIC psychology services; this causes a gap in resourcing, which is subsequently disrupting the regular recording of psychological and rehabilitation outcomes and requires consideration for the embedding of the screening and standards across the NHS England SCICs. In addition to limiting services’ ability to accurately evaluate outcomes, PwSCI/D are at risk of unidentified psychological and/or rehabilitation needs negatively affecting adjustment and transition to community living. 

Allied with this, participants who were not administered psychometric measures at admission were only recorded by the YRSIC sample, and even then, a large number did not provide a reason for the lack of data (*n* = 42). It was not possible, therefore, to determine whether certain groups were at greater risk of being missed, for example, short-stay admissions.

The current findings demonstrated the predictive influence of admission score on discharge score across all three measures. However, the level of injury also had a negative predictive effect on the discharge score for the measures that screened for mood such that people with higher levels of injury had higher depression and anxiety scores at discharge. This is in contrast to previous research that indicated the negligible influence of the level of injury on either depression or anxiety severity [58,59]. As such, the conflicting evidence requires further review.

A key limitation in the current study was that psychological treatment was not able to be controlled for during the analysis due to the retrospective design, which future research should consider. The influence of psychological intervention in relation to improvements in mood and appraisals from admission to discharge would have been useful to examine. Additionally, systematic screening compared with referral data could have further elucidated the need for model change. In addition, though out of scope for the current study, is the consideration of length of stay for individuals who screen positive/receive a psychological intervention, with recent research suggesting comparatively longer hospital admissions for people who screened positive on measures of mood and appraisals compared with those who did not [1]. Additionally, a limitation of the current study, and an area of much-needed research, is to focus not only on screening for those admitted for first-time rehabilitation but also on the needs of those who age with injury [60] and who undergo earlier physical decline associated with their injury than non-injured counterparts [61]. The recommendations of the standards and screening are for across the lifespan, and thus, it is hoped that this will be addressed in time. Indeed, another key area for the future is to survey across the system knowledge and implementation of the standards [62].

While the current findings were important for identifying mood and appraisals for PwSCI/D, future guidance and screening should also examine a range of other psychological concerns, and readers are directed to the work currently being undertaken as part of the ISCoS Advanced Psychological Dataset. The introduction of screening in the NHS England SCICs also included assessing pain, and future research should consider this area alongside mood and appraisals. Had pain data been included in the current sample, it could have illuminated the effect of pain on psychological needs, which would have added to the robustness of the findings. Additionally, this would have enabled clinicians and MDT to be more aware of the complex dynamic between pain and psychological need to better support PwSCI/D during rehabilitation [48].

## 5. Conclusions

This study has provided support for NHS England SCIC psychological services to systematically move to a screen and routine assessment model, with the inclusion of referral options for those whose needs change over time and/or present with difficulties outside the remit of screening. There is a range of evidence from other conditions supporting the advantages of such developments, though the scale of the task and embedding of the standards is significant given the current staffing variation across SCICs. This study also supports the adoption of screening across the system in recognition of the capacity demands in NHS England and the consideration of screening and standards for psychological care by the wider SCIPAG and the worldwide psychology community.

## 6. Patents

The Stoke Mandeville Spinal Needs Assessment Checklist (SMS-NAC) is the intellectual property of and invention of the Department of Clinical Psychology of the National Spinal Injuries Centre, Stoke Mandeville Hospital, Buckinghamshire Healthcare NHS Trust, Aylesbury, UK (UK Copyright Services Registration Number 284732791). The NSIC Psychological Care Pathway is the intellectual property of the Department of Clinical Psychology of the National Spinal Injuries Centre, Stoke Mandeville Hospital, Buckinghamshire Healthcare NHS Trust, Aylesbury, UK (UK Copyright Services Registration Number 284734611), and provided the basis for the Psychological Health and Wellbeing Matched Collaborative Care Intervention Pathway (Supplementary Table S1). Please contact bht.nsicpsychology@nhs.net to seek permission for use.

## Figures and Tables

**Table 1:** Participant demographics and descriptive statistics across samples for all participants.

		N (% Total Number)			
		NSIC	MCSI	YRSIC	Combined
**Total**		438	87	121	646
**Sex**	Male	297 (68)	62 (71)	76 (63)	435 (67)
	Female	141 (32)	25 (29)	45 (37)	211 (33)
**Ethnicity**	White	266 (61)	54 (63)	106 (88) *	426 (66)
	Black	30 (7)	3 (3)	1 (1)	34 (5)
	Asian	24 (5)	1 (1)	6 (5)	31 (5)
	Mixed	4 (1)	1 (1)	1 (1)	6 (1)
	Other	3 (1)	0 (0)	3 (2)	6 (1)
	Not stated	111 (25)	28 (32)	4 (3)	143 (22)
**Cause of injury**	Traumatic	221 (51)	58 (67)	46 (38) *	325 (51)
	Non-traumatic	217 (49)	29 (33)	75 (62)	321 (49)
**Level of injury**	Tetraplegia (A/B/C)	89 (20)	28 (32)	37 (31)	154 (24)
	Paraplegia (A/B/C)	168 (39)	24 (28)	46 (38)	238 (37)
	All levels D	181 (41)	32 (37)	26 (21) *	239 (37)
	Not stated	0 (0)	3 (3)	12 (10)	15 (2)
**Psychometrics**	PHQ-9	432 (99)	85 (98)	115 (95)	632 (98)
	GAD-7	432 (99)	86 (99)	118 (98)	636 (98)
	ADAPSSsf	423 (97)	83 (95)	110 (91)	616 (95)
		**Mean (St. Dev.)**			
		**NSIC**	**MCSI**	**YRSIC**	**Combined**
**Age at injury (years)**		54.64 (17.56) *	58.36 (19.14)	60.23 (16.20)	56.19 (17.66)
**Time since injury (years)**		0.41 (0.42)	0.37 (0.17)	0.21 (0.30) *	0.37 (0.38)
**PHQ-9 total score**		6.38 (5.86)	4.95 (4.81)	7.03 (6.56)	6.31 (5.88)
**GAD-7 total score**		4.44 (5.16)	2.92 (3.70)	4.96 (5.67)	4.33 (5.12)
**ADAPSSsf total score**		19.18 (6.60)	18.78 (6.18)	19.05 (6.59)	19.11 (6.59)

Note: * indicates significance.

**Table 2:** Psychometric properties for combined sample for those with admission measures.

		**Combined Sample N (% Excluding Missing Values)**
**Psychometrics**	PHQ-9	632
	GAD-7	636
	ADAPSSsf	616
**Above threshold**	PHQ-9 (≥11)	138 (22)
	GAD-7 (≥8)	142 (22)
	ADAPSSsf (≥22)	206 (33)
		**Combined Sample Mean (St. Dev.)**
**PHQ-9**	Above threshold	15.54 (3.90)
	Below threshold	3.72 (3.09)
**GAD-7**	Above threshold	12.60 (3.56)
	Below threshold	1.95 (2.18)
**ADAPSSsf**	Above threshold	26.50 (3.53)
	Below threshold	15.39 (4.20)

**Table 3:** Participant PHQ-9, GAD-7 and ADAPSSsf score across samples for those with admission and discharge measures.

	Mean (St. Dev.)			
	NSIC	MCSI	YRSIC	Combined
**PHQ-9 total—admission**	6.26 (5.73)	5.41 (5.21)	8.23 (6.95)	6.33 (5.80)
**PHQ-9 total—discharge**	5.35 (5.98)	2.89 (3.41)	6.30 (5.46)	5.19 (5.77)
**GAD-7 total—admission**	4.23 (5.14)	3.75 (4.67)	5.22 (6.19)	4.27 (5.18)
**GAD-7 total—discharge**	3.84 (5.09)	2.59 (4.03)	4.04 (3.07)	3.74 (4.85)
**ADAPSSsf total—admission**	19.44 (6.77)	20.42 (6.31)	18.55 (7.13)	19.46 (6.75)
**ADAPSSsf total—discharge**	19.28 (6.47)	17.50 (5.83)	18.14 (6.80)	19.01 (6.44)

**Table 4:** PHQ-9 and GAD-7 severity classifications for combined sample for those with admission and discharge measures.

		Combined Sample N (%)	
		Admission	Discharge
**PHQ-9 severity**	**Total N**	**266**	**270**
	Sub-clinical	126 (47)	157 (58)
	Mild depression	71 (27)	63 (23)
	Moderate depression	41 (15)	29 (11)
	Moderately severe depression	18 (7)	11 (4)
	Severe depression	10 (4)	10 (4)
**GAD-7 severity**	**Total N**	**267**	**269**
	Sub-clinical anxiety	174 (65)	181 (67)
	Mild anxiety	44 (17)	50 (19)
	Moderate anxiety	32 (12)	26 (10)
	Severe anxiety	17 (6)	12 (4)

**Table 5:** Psychometric properties for combined sample for those with admission and discharge measures.

		**Combined Sample N (% Excluding Missing Values)**	
		**Admission**	**Discharge**
**Psychometrics**	PHQ-9	266	270
	GAD-7	267	269
	ADAPSSsf	267	267
**Above threshold**	PHQ-9	61 (23)	38 (14)
	GAD-7	60 (23)	44 (16)
	ADAPSSsf	98 (37)	94 (35)
		**Combined Sample Mean (St. Dev.)**	
		**Admission**	**Discharge**
**PHQ-9**	Above threshold	15.11 (3.83)	16.68 (4.65)
	Below threshold	3.72 (3.08)	3.31 (3.17)
**GAD-7**	Above threshold	12.63 (3.63)	12.89 (4.05)
	Below threshold	1.84 (2.15)	1.95 (2.31)
**ADAPSSsf**	Above threshold	26.49 (3.73)	25.72 (3.63)
	Below threshold	15.38 (4.30)	15.36 (4.37)

**Table 6:** Multiple regression on the predictive relationship between admission scores and demographic variables on discharge score for the PHQ-9, GAD-7 and ADAPPsf.

				95% CI			
		Beta	*SE*	LB	UB	β	*p*
**PHQ-9**	Admission score	0.493	0.052	0.390	0.596	0.493	<0.001 *
	Level of injury	−1.243	0.410	−2.051	−0.435	−0.160	0.003 *
	Age at injury	−0.053	0.019	−0.090	−0.016	−0.154	0.005 *
	Ethnicity	−0.135	0.145	−0.422	0.151	−0.049	0.352
	Cause of injury	0.379	0.609	−0.820	1.577	0.033	0.534
	Time since injury	0.348	0.844	−1.315	2.011	0.022	0.681
	Sex	−0.090	0.662	−1.393	1.213	0.007	0.892
**GAD-7**	Admission score	0.473	0.048	0.379	0.567	0.504	<0.001 *
	Level of injury	−1.172	0.330	−1.822	−0.522	−0.181	<0.001 *
	Age at injury	−0.054	0.015	−0.084	−0.024	−0.186	<0.001 *
	Ethnicity	−0.072	0.118	−0.304	0.160	−0.031	0.541
	Cause of injury	0.533	0.491	−0.435	1.501	0.055	0.279
	Time since injury	0.724	0.688	−0.630	2.078	0.053	0.293
	Sex	0.245	0.532	−0.802	1.292	0.023	0.645
**ADAPPsf**	Admission score	0.574	0.047	0.481	0.667	0.604	<0.001 *
	Level of injury	−0.768	0.434	−0.89	−1.622	0.086	0.078
	Age at injury	0.004	0.020	−0.035	0.043	0.010	0.839
	Ethnicity	0.220	0.153	−0.81	0.521	0.072	0.152
	Cause of injury	0.028	0.641	−1.235	1.291	0.002	0.965
	Time since injury	0.991	0.896	−0.773	2.755	0.056	0.270
	Sex	0.012	0.687	−1.341	1.366	0.001	0.986

Note: * indicates significance.

## Data Availability

The data used in the current study were part of a standard clinical database that contains identifiable patient information and are, therefore, not publicly available. Anonymised data can be made available upon reasonable request to Buckinghamshire Healthcare NHS Trust.

## Supplementary Materials

The following supporting information can be downloaded from https://www.mdpi.com/article/10.3390/jcm12247667/s1, Figure S1: Preadmission Outreach Pathway; Table S1: Psychological Health and Wellbeing Matched Collaborative Care Intervention Pathway; Table S2: SCI MDT Education Curriculum.

## Abbreviation

Acronym description and short explanation.

**Acronym**	**Meaning**
ADAPPSsf	Appraisals of DisAbility Primary and Secondary Scale short form. This is a six-item scale that assesses a participant’s appraisal of their injury and provides an indication of adjustment to SCI/D and screens for a full-scale version
CPG	Clinical Practice Guideline
CRG	Clinical Reference Group, which oversees the service provision for a range of health conditions for NHS England and sets the service specification and standards for care
EBM	Evidence-based medicine
GAD-7	General Anxiety Disorder-7 is a seven-item measure used to assess the presence of anxiety symptoms
ISCoS	The International Spinal Cord Society
MCSI	Midlands Centre for Spinal Injuries
MDT	Multidisciplinary Team
NHS	National Health Service, which is the universal health care provider in the UK; the NHS provision is devolved into separate bodies for England, Wales, Scotland and Northern Ireland
NICE	National Institute for Health and Care Excellence is an overarching body that creates recommendations for treatment protocols and medication
NSIC	National Spinal Injuries Centre at Stoke Mandeville
PHQ-9	Patient Health Questionnaire is a nine-item measure used to assess the presence of depression symptoms
PwSCI/D	People with spinal cord injuries/disorders
SCI/D	Spinal cord injuries/disorders
SCICs	Spinal cord injury centres provide inpatient rehabilitation in the UK
SCIPAG	UK and Ireland Spinal Cord Injury Psychology Advisory Group review and promote psychological service provision and care standards across the SCICs
SIG	Special interest group
SMS-NAC	Stoke Mandeville Spinal Needs Assessment Checklist, which is used to assess inpatient’s knowledge, skills, and physical or verbal independence across 10 domains of rehabilitation for SCI/D
YRSIC	Yorkshire Regional Spinal Injuries Centre

## Appendix A. Standards Recommendations for System-Wide Change with Table

The below information outlines the mental health standards for implementation in UK Spinal Cord Injury Services. Table A1 also highlights the timeline for implementation of the standards and relevant citations.

Standards:

1. Adoption across all providers of a matched collaborative care pathway (see also Supplementary Table S1). The NSIC Stoke Mandeville Psychological Care Pathway (UK Copyright Service 284734611) proposed foundation needs for all PwSCI/D and identified the need for a clinician and peer-facilitated coping effectiveness group intervention to aid self-management, alongside psychoeducation and consideration of psychosexual and family counselling, with four specific interventions depending on needs [63]. The workstream enhanced this by adding screening thresholds for the interventions and renamed it the “psychological health and wellbeing matched collaborative care intervention pathway” to aid system-wide adoption and comparison between SCICs and others in the pathway regarding complexity and workforce need.
2. Adoption across all providers of an MDT curriculum, with basic (Level 1) skills needed by all healthcare professionals who have contact with PwSCI/D, with someone trained to advanced (Level 2) skills within each team/clinical area (Supplementary Table S2).
3. A preadmission outreach pathway to ensure the parity of admission for people with complex mental health needs (Supplementary Figure S1).
4. Implementation of psychological health screening on admission, discharge, readmission and outpatient review across the sector.
5. The workstream acknowledged the variety of resourcing across the SCICs and other providers as a limiting factor for the implementation of the standards. Therefore, it was recommended that all services be resourced similarly and to at least the staffing of the current best ratio (London SCIC 1:15) and/or aligned with other SCI providers in the network, such as neurorehabilitation services given the complexity of need [64]. The workstream anticipated that some services would be nonadherent because of staffing variation and recommended yearly audits by SCIPAG/peer review, and where service gaps are identified, an action plan should be implemented.
6. The workstream recommended support to consult, finalise and publish the broader evidence-based standards that had been commenced by SCIPAG.
7. Three key areas for development were identified across the sector:
  i. SCIC Outpatient Services—The workstream referenced the need for psychological support for adjustment to injury to be about 40% (not including those referred to community mental health services) and noted the high prevalence of persistent pain and the current gap in services for PwSCI/D [50,65]. The workstream recommended the development of an MDT clinic and estimated that 60–70% of people presenting with persistent pain would need an associated psychological review.
  ii. Traumatic Brain Injury (TBI) provision within SCICs. The workstream acknowledged that whilst SCICs manage the needs of those with co-morbid mild TBIs, those with more severe injuries often fall between neurorehabilitation and SCI/D rehabilitation services. Recommended future development should focus on (a) scoping local services and developing links, providing an integrated pathway for those with moderate TBI and SCI/D by embedding neuro-rehabilitation expertise within SCICs and vice versa, including joint training events and rotational arrangements for therapists and nurses in the first instance, and (b) progress to the employment of staff skilled in managing moderate TBI in SCICs.
  iii. Psychiatry provision. The workstream recommended the following: (a) services should foster links with local specialist mental health services, particularly liaison psychiatry services; (b) have service level arrangements with or embed liaison psychiatry services within SCI/D services; (c) improve the training of staff to better manage mental health complexity on SCI/D units through the adoption of the MDT curriculum identified (Supplementary Table S2); (d) arrange collaborative and parallel working practices for people with co-occurring complex mental health and spinal cord injury rehabilitation needs, such as repatriation arrangements; (e) agree on responsible clinician arrangements with local specialist services for people detained under the Mental Health Act [66]; and (f) the development of wheelchair-accessible services across mental health units for PwSCI/D.

**Table A1:** Quality standards summary and timings for implementation.

Timing	Quality Standard Summary	References
**Onset of injury/acute care**	Psychological health screen (PHQ-5) within 4 weeks of injury.	[14,18,64,67,68]
	Assessment prior to SCIC/rehabilitation transfer to include screening measures and structured clinical interview with information about known mental health and forensic history, any barriers or additional needs for engagement in rehabilitation, and past and present mental health professional involvement. To be completed where relevant: MOCA, AMTS, 6CIT or another recognised cognitive test if the person has a pre-existing or current cognitive impairment. An assessment of alcohol, tobacco, and recreational drug history and current use. A mental capacity assessment.	[14,18,67,69,70]
	Amendment of the NHS England Database to recategorise the current category “mental health” and instead categorise using self-harm/suicide attempt/neglect, severe and enduring mental health/psychosis/schizophrenia, depression/anxiety, substance use, neurodevelopmental diagnosis or dementia.	-
	Implement pre-admission outreach flowchart across SCICs and yearly audit of its use by SCIPAG/peer review.	-
**Admission to SCIC/rehabilitation**	All inpatients to have access to specialist evidence-based psychological treatment intervention and include trauma-based intervention.	[14,15,16,17,18,20,25,26,63,64,67,68,70,71,72,73,74]
	Implementation of the psychometric screening measures across all parts of the pathway and for all levels and completeness of SCI/D.	[14,17,18,67]
	Implementation of the Psychological Health and Wellbeing Matched Collaborative Care Intervention Pathway (Supplementary Table S1) across SCICs and service provision alignment. Yearly audit of implementation and complexity of inpatient needs.	[63,69,71]
	Documented pathway for access to liaison psychiatry and other specialist services.	[14,18,20,25,26,63,64,67,69,73,75,76,77]
	Outcome comparison by SCIC and other services to track group trajectory profiles by complexity with revision of pathway as required.	-
	Initial contact from a psychosocial team member within 5 days of inpatient admission.	[14,18,67,71]
	Inpatient access to specialist psychological assessment and therapy within 10 working days of admission and include psychological health screen with psychometrically validated tools.	[14,15,16,18,20,63,67,69,70,73,75,76,77,78]
	Where suicidality is present, risk assessment, personal safety plan and treatment plan are to be established.	[14,18,48,67,73,77]
	Where motivation/engagement/progress in rehabilitation is limiting progress/change, psychological assessment and intervention should be provided.	[14,15,18,71,75]
	Peer support and peer mentoring should be available for all inpatients and the psychosocial care team leading on recruitment and organisation of this model within the SCIC.	[18,20,25,26,63,64,67,70,73]
	Provision of support services for the psychological/emotional needs of families/carers, including referrals.	[18,25,26,63,64,69,71,73]
	Adoption of the SCI MDT Education Curriculum (Supplementary Table S2) to align healthcare clinicians working in SCICs to be able to identify and support patients’ psychosocial needs, e.g., mood, adjustment issues, risk, substance use, cognition, and behaviours that challenge and know how to escalate for specialist psychological intervention as needed. All staff to have basic (Tier 1) skills and some staff to have advanced (Tier 2) skills.	[14,18,63,67,69,71,73,78]
	Inpatients, families and carers are to be offered support on self-management skills and empowered to advocate for their needs and seek support.	[15,18,64,67,71,73,76,78]
**Discharge** **from rehabilitation**	Psychological health psychometric screening and psychological assessment are to be repeated prior to discharge.	[14,18,25,26,67,69,73,78]
	Comprehensive psychological discharge planning, including referrals to relevant services.	[14,18,25,26,67,69,73]
	Onward referral made as required to the BackUp Trust peer mentoring or Spinal Injuries Association counselling service.	[18,64]
**Follow-up**	Psychological health screen (PHQ-4) and substance/alcohol use screen to take place across four time points/ranges: 6–12 weeks post-discharge, 6 months, annually for 5 years, and then every 2 years or as required.	[14,16,18,25,26,64,67,69,70,78]
**SCIC/** **hospital readmission**	All secondary rehabilitation admissions of PwSCI/D are to be administered a short form psychological health screen (PHQ-4). A referral is to be made to the SCIC psychological services for full assessment if there is a positive screen.	[14,18,67,71,73,77]
