# Supplementary material for: Psychological Screening, Standards and Spinal Cord Injury: Introducing Change in NHS England Commissioned Services
Source: J Clin Med. 2023 Dec 13;12(24):7667. doi: 10.3390/jcm12247667 (PMC10743880; doi:10.3390/jcm12247667)
Supplement: Supplementary file 1 [file jcm-12-07667-s001.zip › jcm-2718484-supplementary.pdf]

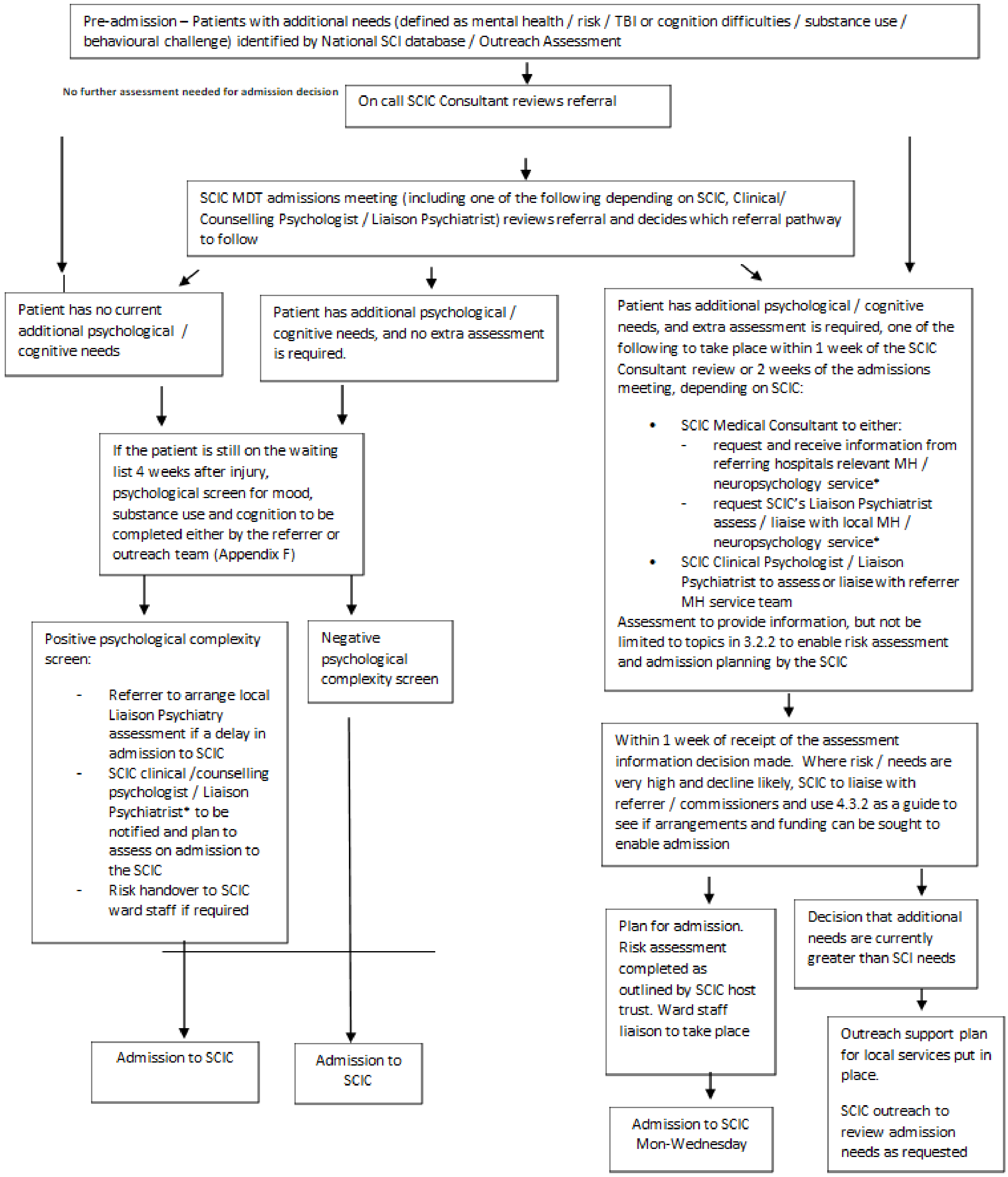

*Relevant MH service will in most instances be the referrer's Liaison Psychiatry service, but could instead include depending on local arrangements the referrers clinical psychology / or health psychology service

Figure S1. Preadmission Outreach Pathway.

Table S1. Psychological Health and Wellbeing Matched Collaborative Care Intervention Pathway. A complexity pathway was developed and copyrighted by the National Spinal Injuries Centre Stoke Mandeville following the tariff review in 2014 (UK Copyright Services Registration Number 284734611) which had been adopted by some SCICs. The workstream revised the pathway and proposed possible preliminary screening thresholds, the workstream recommends its introduction across inpatient SCICs.

	**Clinical Presentation**	**Preadmission Outreach and previous Mental Health (MH)**	**Psychological Therapy Contact**	**MDT Skills and Consultation**	**Referral / treatment from Specialist co-located service for additional need**	**Keyworker and Goal Planning**	**Discharge Planning**
**1 BRIEF INTERVENTION**	Past:<br><br>No previous MH or previous MH needing intervention in primary care<br><br>Present:<br><br>Predominant presentation of symptoms below clinical threshold for depression/ anxiety or adjustment<br><br>Subthreshold Sx of mood or anxiety<br><br>(PHQ9 <10; GAD7 <10)<br><br>Some difficulty coping – ADAPSSsf profile to guide intervention<br><br>Well-circumscribed and understandable anxieties e.g., fear of falling when transferring	May have had positive Outreach screen. SCIC extended preadmission liaison not usually required.	Initial assessment and treatment intervention usually time limited e.g., 1-3 sessions, then may become periodical and relating to rehabilitation concerns.<br><br>Group intervention will augment individual.	Level 1 MDT psychological care skills.<br><br>Consultation usually takes place as standard in planned MDT meetings and needs review through attendance at goal planning meetings. Extended consultation not usually required.	Not usually required	MDT member Keyworker<br><br>Usual frequency of goal planning meetings	May need onward referral via GP to IAPT or mentoring support<br><br>Contact and discharge summary on IDR

	No active suicidal ideation or self-harm risk, passive suicidal ideation may be present<br><br><u>Cognitive Issues</u>:<br><br>No cognitive issues						
**2**<br><br>**ROUTINE INTERVENTION**	<u>Past</u><br><br>No previous MH or previous MH needing intervention in primary care<br><br>Other pre-morbid condition such as learning disability or dementia or current co-morbid condition such as TBI which complicates adjustment<br><br>And / or above in association with:<br><br><u>Present:</u><br><br>Predominant presentation of symptoms above clinical threshold for depression/ anxiety or adjustment<br><br>Mild Sx of mood or anxiety disorder, above threshold (PHQ9 10-15; GAD7 11-14)	May have had positive Outreach screen. SCIC extended preadmission liaison not usually required unless screen identified additional needs that could impact on rehabilitation engagement e.g., TBI, substance use	Regular individual treatment intervention, following assessment which is augmented by group intervention.<br><br>Cognitive assessment needed for positive screen and management advice provided to the team.	Level 2 MDT psychological care skills.<br><br>Consultation usually takes place as standard in planned MDT meetings and needs review through attendance at goal planning meetings. So me minimum extended consultation might be required.	Consultation may be required e.g., discussion with liaison psychiatry / substance use where non-response to interventions e.g. PTSD or if complex discharge needed and to minimise risk of relapse of alcohol / drug misuse	MDT member most usually Keyworker or may be SCIC psychologist if interpersonal issues affect adjustment / supervision provided by SCIC psychologist if not Keyworker.<br><br>Usual frequency of goal planning meetings	May need onward referral via GP to IAPT or mentoring support<br><br>Contact and discharge summary on IDR

	ADAPSSsf profile to guide intervention, may need full scale ADAPSS<br><br>Symptoms of PTSD may be present<br><br>No active suicidal ideation or self-harm risk, passive suicidal ideation may be present<br><br>Pre-injury regular excessive alcohol use or dependence<br><br><u>Cognitive Issues</u>:<br><br>Mild cognitive impairment impacting upon rehabilitation e.g., difficulty with SCI education or carry-over between PT/OT sessions<br><br>Borderline intellectual disability (IQ >70; mental age of 12) impacting upon rehabilitation; or mild intellectual disability (IQ >50, mental age of 9-12)						
**3**	May have previous contact with MH / GP	Positive Outreach screen including previous MH	Significant individual treatment intervention,	Level 2 MDT psychological care skills.	<u>Psychiatric</u>	SCIC Psychologis t Keyworker	Significant discharge planning and liaison which

C<br>O<br>M<br>P<br>L<br>E<br>X	services or other services for pre-morbid condition<br><br>History and risk (but no active or recent presentation) of self-harm or imminent risk to self or others; and / or chronic mental health difficulties with acute relapse; active issues with substance use;<br><br>severe interpersonal difficulties / behaviours that challenge<br><br>Risk of relapse and / or<br><br>self-neglect<br><br>Moderate Sx of mood or anxiety disorder (PHQ9 15-20; GAD7 15-18)<br><br>ADAPSSsf profile to guide intervention, may need full scale ADAPSS<br><br>Ongoing alcohol misuse or craving for alcohol; H/O regular substance misuse; evidence of prescribed drug addiction	needs identified. SCIC extended preadmission liaison required which may include requesting local liaison psychiatric report / liaison with previous CMHT contact / neuropsychological screening leading to SCIC planning care such as personal safety planning.	following assessment which is augmented by group intervention.<br><br>SCIC psychologist to provide or refer for extended neuropsychological assessment. SCIC psychologist to provide management advice to the team.	Significant consultation required outside of MDT and goal planning meetings.<br><br>SCIC psychologist actively involved in team risk management/ safeguarding and is link for liaison with MH services can be required to provide consultation in crisis situations. Risk managed through psychological consultation with team and provision of adequate support structure.	Significant liaison including:<br><br>- preadmission consultation as required<br><br>- regular risk review including safety netting and medicines optimisation.<br><br>Substance Use – Intervention as per local service<br><br>Neurology / Dementia care<br><br>– Assessment and intervention as per local service<br><br>NeuroPsychologist<br><br>– Assessment and intervention as per local service<br><br>Other services may be required.	/ supervision provided by psychologist if MDT member is the Keyworker.<br><br>Goal planning meetings usual intensity, may include support in between	may include active psychiatric involvement to ensure smooth handover to community services.<br><br>Discharge Letter written with recommendations and onward referral

	Stable but serious mental illness: schizophrenia, bipolar affective disorder, eating disorder, personality disorder<br><br><u>Cognitive Issues</u>:<br><br>Significant cognitive difficulties eg. 6CIT ≤ 8 or AMTS < 8 or MOCA <20; in people with tetraplegia: MOCA-Blind <15<br><br>Moderate intellectual disability (IQ >35; mental age of 6)						
**4**<br><br>**HIGHLY COMPLEX**	Likely to have previous substantial contact with MH or other services for pre-morbid condition<br><br>Recent /active self-harm or risk (which could be imminent) to self or others; chronic mental health difficulties with acute relapse; active issues with substance use; behaviours that challenge<br><br>severe interpersonal difficulties/those with high levels of social deprivation which affects patient	Positive Outreach screen including previous MH needs identified. SCIC extended and substantial preadmission liaison required which may include requesting local liaison psychiatric report / liaison with previous CMHT contact / neuropsychological screening leading to SCIC planning care such as personal safety planning.	Substantial and frequent individual treatment which at times of crisis may be more often than once a week.<br><br>SCIC psychologist to provide or refer for full neuropsychological assessment. SCIC psychologist to provide management advice to the team.	Level 2 MDT psychological care skills.<br><br>Substantial and frequent consultation outside of MDT and goal planning meetings. Often weekly or more frequent at times of crisis.<br><br>SCIC Psychologist leads in team risk management/ safeguarding is link for liaison with MH services and team requests consultation in crisis situations	<u>Psychiatric</u><br><br>-substantial, active and regular liaison response.<br><br>-medicines management<br><br>-active risk management<br><br><u>Substance Use</u> – Intervention as per local service<br><br><u>Neurology / Dementia care</u><br><br>– Assessment and intervention as per local service<br><br><u>NeuroPsychologist</u><br><br>– Assessment and intervention	SCIC Psychologist Keyworker / supervision provided by SCIC psychologist if MDT member is the Keyworker.<br><br>Goal Planning meetings often more frequent including liaison in between meetings	Complex, substantial liaison with community staff pre discharge and may include active psychiatric involvement to ensure smooth handover to community services.<br><br>Discharge Letter written with recommendations and onward referral

	engagement and safety.<br><br>Severe Sx of mood or anxiety disorder (PHQ9 >20 or GAD7 >18)<br><br>ADAPSSsf profile to guide intervention, may need full scale ADAPSS<br><br>History of injecting drug use; history of substance dependence; methadone prescription<br><br>Unstable serious mental illness<br><br><u>Cognitive Issues:</u><br><br>Delirium not responding to standard treatment				as per local service<br><br>Other services may be required.		

Table S2. SCI MDT Education Curriculum. The workstream used the Psychological Health and Wellbeing Matched Collaborative Care Intervention Pathway, Supplementary Table S1 to develop a SCI specific curriculum for MDT staff, identifying basic (Level 1) skills that are needed by all staff working in the SCIC, with some staff in each clinical area needing advanced (Level 2) skills. The SCI MDT curriculum recognises the psychological/mental health first aid and generic emotional skills support provided by members of the MDT, which complements the specialist individual and group psychotherapy provided in the matched collaborative care assessment and treatment framework, Supplementary Table S1. The below curriculum draws on and includes the Mental Health Core Skills Education and Training Framework, (Skills for Health and Mental-Health-CSTF.pdf).

Level 1		
**Subject**	**Content**	**Objectives**
Being in hospital	• The meaning of hospital for patients<br>• What a hospital can look and feel like to patients<br>• The effects of the above<br>• From person to patient (pros and cons)<br>• Helping a patient to maintain their identity<br>• Why sleep is important<br>• The normal sleep cycles<br>• Effect of admission to hospital on sleep<br>• Sleep strategies for the patient<br>• What night staff can do to help<br>• Cognitive Fatigue / fatigue and SCI<br>• Active listening skills – hearing what is beyond the statement and how to respond	• To understand how hospitalisation can affect a patient and their family<br>• To understand sleep, be able to support the patient to improve their sleep and change own behaviour that might be affecting the patient's sleep
Interaction between physical and mental health (biopsychosocialspiritual model)<br><br>*Understanding the patient (simple formulations)*	• What is the biopsychosocial spiritual model?<br>• Why it is important to consider in the hospital ward<br>• Mood and thoughts can affect physical symptoms<br>• Is the patient exaggerating or could it be something else?<br>• What is a formulation<br>• Why are they important when working with a patient?<br>• How you can use them in your work with patients<br>• A framework for understanding diagnosis, prognosis and common reactions<br>• The role of hope (that it is not denial) and pendulum that people experience as they approach what SCI means, back away (avoid) and then approach again<br>• Context and coping with behaviours that challenge<br><br>• **Mental Health Core Skills Education and Training Framework** Subject 12 and Ref: Y/602/6374 Level 1 Introduction to mental health<br>• Violence and aggression: short-term management in mental health, health and community settings (nice.org.uk)	• To understand the biopsychosocial spiritual model and the implications for working with patients<br>• To recognise that every patient is different and therefore treated as individuals and without judgement<br>• To understand what a formulation is; its role and how to develop and communicate a basic formulation
Depression	• What is depression?<br>• Causes of depression<br>• Symptoms of depression<br>• Effects of depression<br>• Working with the patient who is depressed<br><br>• **Mental Health Core Skills Education and Training Framework** Subject 4, 5, 11, 13 and Ref: Y/602/6374 Level 1 Introduction to mental health	• To understand depression, its causes and effects.<br>• To feel confident working with a patient who is depressed
Anxiety	• What is anxiety?<br>• Causes of anxiety<br>• Symptoms of anxiety<br>• Effects of anxiety<br>• Working with the patient who is anxious	• To understand anxiety, its causes and effects.<br>• To feel confident working with a patient who is anxious

	• **Mental Health Core Skills Education and Training Framework** Subject 4, 11, 13 and Ref: Y/602/6374 Level 1 Introduction to mental health	
Risk assessment	• What is self-harm and what is suicidal intent<br>• How do you know a patient is suicidal?<br>• Assessing a suicidal patient<br>• The words to use<br>• Action to take<br>• The myth that talking to a suicidal patient will make it more likely that they will attempt to end their life<br>• Effect on you when a patient is suicidal & how to manage these feelings and afterwards<br><br>• **Mental Health Core Skills Education and Training Framework** Subject 2, 3, 5, 7, 11, Ref: R/602/6194 Level 1 Awareness of protection and safeguarding in health and social care (adults and children and young people), early years and childcare and Ref: A/601/8574 Level 2 Principles of safeguarding and protection in health and social care	• To be able to assess a patient who is thought to be at risk of suicide<br>• To be able to respond with appropriate actions to ensure the safety of the patient<br>• To be able to recognise and understand the impact on the clinician and to ensure self-care
Pain on the ward	• What is pain (biopsychosocial and basic pain mechanisms)?<br>• Acute and chronic pain<br>• Culture and pain and expression of pain<br>• What can increase a patient's pain<br>• Can we tell how much pain patients are in?<br>• How to assess pain<br>• How to respond to pain<br><br>• **Mental Health Core Skills Education and Training Framework** Subject 3	• To have a basic understanding of pain mechanisms<br>• To understand the difference between acute and chronic pain<br>• To understand that pain is always a mix of physical and psychological<br>• To understand what affects pain<br>• To be able to assess and respond to patients' pain
Cognition	• Memory, attention, dysexecutive difficulties<br>• What to look for in rehab	• To be able to recognise signs of memory, attention, dysexecutive difficulties<br>• To know how to refer<br>• To know how to make basic adaptations to a care environment
Alcohol and substance use	• What this is alcohol and substance use and how to recognise<br>• Behaviours that may be associated with this<br>• Different approaches abstinence/relapse prevention<br>• When you feel conflicted e.g., smoking/bed rest, boundaries when someone tells you about substance use<br><br>• **Mental Health Core Skills Education and Training Framework** Subject 13	• To have a basic understanding of alcohol and substance use and what this looks like in rehab environment<br>• To practice verbal replies to verbal and physical behaviours that challenge, role of consistency across MDT
Severe and enduring mental health	• Basic MH knowledge and awareness<br>• Behaviours that might be associated with this<br>• Taboos and myths<br>• Risk Assessment<br>• Basic information and limits on the Mental Health Act, holding powers etc<br>• **Mental Health Core Skills Education and Training Framework** Subject 6, 7, 13, 16	• To have a basic understanding of MH needs and what this looks like in rehab environment<br>• To know how to adapt the care environment/intervention/therapy to accommodate<br>• To be able to have a risk conversation
Culture and language	• Different language and use of interpreters<br>• Different culture<br>• Same language, different culture<br><br>• **Mental Health Core Skills Education and Training Framework** Subject 5 and Ref: H/602/3039 Level 2 Principles of diversity, equality and inclusion in adult social care settings	• To have attended local Trust level awareness<br>• To be aware of local SCIC demographics and need<br>• Unconscious bias and inclusion examples

Managing difficult interactions with patients and families	• Understanding the patient and the family and their stress/mood<br>• What is anger and how much anger do we tolerate<br>• Noticing an escalating situation<br>• What helps and what does not help<br>• Managing your own reactions to others' stress<br>• Difficult conversations feel difficult, this is normal<br>• Plan (but don't over plan) what you need to say<br>• Spend some time reflecting on how the subject affects you<br>• Confidential, uninterrupted space<br>• Consider environment (e.g., no desk between you; same height chairs)<br>• You and the patient must have plenty of time<br><br>• **Mental Health Core Skills Education and Training Framework** Subject 8 and 9<br>• Violence and aggression: short-term management in mental health, health and community settings (nice.org.uk)	• To be able to have an effective difficult conversation with a patient/their family
Self-management and adjustment model	• Motivation for goals and what helps people to adhere<br>• Specific goals / targets, who, what, where, when<br>• Goals and care planning<br>• Participation and impact on adjustment – work, quality of life, implicit expectations<br><br>• **Mental Health Core Skills Education and Training Framework** Subject 14<br>• https://skillsforhealth.org.uk/wp-content/uploads/2021/01/Person-Centred-Approaches-Framework.pdf<br>• https://www.england.nhs.uk/wp-content/uploads/2019/01/universal-personalised-care.pdf<br>• Violence and aggression: short-term management in mental health, health and community settings (nice.org.uk)	• Be aware of basic principles of adherence, factors that facilitate and diminish it<br>• Have basic knowledge of motivational interviewing/health coaching and how to engage people in positive decision making<br>• Be able to set specific goals and targets in own clinical area
Sexuality	• PLISSIT model (Permission, Limited Information, Specific Suggestions, Intensive Therapy – skilled in P and LI)<br>• Basic sexual responsiveness by level of SCI<br>• Signposting people to more specialist support<br>• Understanding of how to apply the clinical practice guide - Sexuality and Reproductive Health in Adults with Spinal Cord Injury: a clinical practice guideline for health professionals (2010). Consortium of Spinal Medicine and Paralyzed Veterans of America	• To have basic knowledge of physical sexual responsiveness by level of injury<br>• To be confident in how to answer a question about options available<br>• To be able to signpost to services
Self-care	• Stress, depression, anxiety<br>• Busy job and burn out<br>• Unhelpful responses and behaviours<br>• Helpful responses and behaviours<br>• When to get more support<br>• Mentoring and clinical supervision - how to use	• To be able to identify signs and symptoms of stress and be aware of triggers<br>• To be aware of and able to discuss impact of a clinical case on own self care
**Level 2**		
**Subject**	**Content**	**Objectives**
A framework for understanding the patient	• Understanding the patient: CBT principles<br>• Hot cross bun (thoughts; emotions; behaviour; physical symptoms)<br>• Hot cross bun and the patient/relatives<br>• Understanding a patient's responses: Examples of patients' hot cross bun<br>• Understanding 'odd' behaviour: Examples<br>• Recognising a downward spiral / unhelpful thoughts	• Have a basic understanding of the relationship between thoughts, emotions, behaviour and somatic symptoms/feelings<br>• Understand what could be underneath / driving patients 'odd' or unwanted behaviour<br>• Develop basic skills in helping patients to consider alternative thoughts
Self-management (patient)	• Medical vs self-management model<br>• Values<br>• Kolb's Learning Cycle<br>• Integrating patient education with SMART goals	• To be able to help the patient set SMART goals<br>• To understand what values are and how they are related to goal setting

	• https://skillsforhealth.org.uk/wp-content/uploads/2021/01/Person-Centred-Approaches-Framework.pdf<br>• https://www.england.nhs.uk/wp-content/uploads/2019/01/universal-personalised-care.pdf	
Cognition	• How to recognise delirium<br>• Mental Capacity Assessment (MCA)<br>• Memory, attention, dysexecutive difficulties – what to look for in rehab and how to adapt the environment<br><br>• **Mental Health Core Skills Education and Training Framework** Subject 16	• To be able to adapt care environment to meet cognitive needs<br>• To be aware and able to write personal safety plan<br>• To be able to complete a MCA
Screening for psychological distress and how to support other staff	• Mental Health First Aid principles<br>• Reflective practice and supervision	• To be able to signpost staff<br>• Co-facilitate reflective practice session with a qualified psychological practitioner taking the lead
Sexual Health	• PLISSIT model (Permission, Limited Information, Specific Suggestions, Intensive Therapy – skilled in P and LI as level 1 (basic) and SS and level 2 (advanced)<br>• Basic sexual responsiveness by level of SCI<br>• Signposting people to more specialist support<br>• Understanding of how to apply the clinical practice guidelines - Sexuality and Reproductive Health in Adults with Spinal Cord Injury: a clinical practice guideline for health professionals (2010). Consortium of Spinal Medicine and Paralyzed Veterans of America	• To have basic knowledge of physical sexual responsiveness by level of injury<br>• To be confident in how to answer a question about options available<br>• To be able to signpost to services<br>• To be able to make specific recommendations and support patient to trial these
Team working in MDT	• Hot Cross Bun and you/the team<br>• Individual clinician's vs MDT vs IDT: What are they and pros and cons<br>• What causes problems in a team<br>• Signs when things aren't working<br>• What to do when things aren't working (including support available in the Trust)<br>• Difficult conversations in a team<br>• Signs when a team is working well<br>• What helps a team to function well?<br>• Hierarchies, respect & opinions	• To be able to co-facilitate team discussion with a qualified psychological practitioner taking the lead
